# Abstract of the Proceedings of the Buffalo Medical Association

**Published:** 1861-12

**Authors:** J. F. Miner

**Affiliations:** Secretary


ART. II.—*Abstract of the Proceedings of the Buffalo Medical Association.*

TUESDAY EVENING, Nov. 5, 1861.

The Association met at the usual hour. Present—Dr. James P. White, Vice President, in the Chair, Drs. Samo, Eastman, Rochester, Ring, Kempson, Johnson, Miner.

Minutes of the last meeting read and approved.

*Dr. Miner*, as substitute for Dr. Whitney, and by his request, proposed Dr. E. P. Gray for membership.

Voluntary communications being in order, upon being called upon, *Dr. Kempson* remarked, that he had come rather for the purpose of listening than of making any communication. Thanked the Association for the courtesy extended to him, and assured the Society that he should take great pleasure in associating with members of the profession in Buffalo, since located as he was, at Fort Erie, C. W., he had little opportunity of professional intercourse elsewhere, and regarded Buffalo as not only their metropolis of commerce, but also of medicine.

*Prof. Rochester* related the following case of apoplexy: A gentleman from the country, fifty years of age, visited a sick relative in the city, and was constantly engaged in the care of his sick friend, for a week or more, taking very little food or rest.

On Friday of last week he visited Dr. R. in his office, in apparently his usual health; returned home, completed some letters which he had been writing, when suddenly he clasped his head with his hands, saying, "Oh ! my head." He soon became partially comatose, but would reply to questions. The pain complained of, was in the left side of the head, paralysis was of the right side.

Warmth was applied and counter irritation. Croton oil administered, which operated very well; coma increased and he died last evening. Regarded this case as an illustration of the effects of exhaustion and fatigue. This patient had none of the precursory indications of such an attack, had enjoyed usual good health; heart and other organs were healthy so far as ascertained. Dr. R. spoke of the general practice of active depletion in apoplexy, making the distinctions which should be observed in the different forms of the disease, and indicating the more rational treatment.

*Dr. Miner* related the following case which he had recently visited in the country, which exhibited evidences of effusion, complicated and modified by some nervous affection resembling more the convulsion of epilepsy than any other disease.

*August 2d*, 1861.—Visited C. S. aged 35, of healthy robust constitution, by occupation a farmer, and accustomed to active labor. Ten years since, he was injured by the falling upon his chest of a barrel of salt, since which time, after unusual fatigue or exposure, has had a peculiar spasm, involving the whole system, and appearing in many respects like epilepsy. The day previous to this visit, had suddenly fallen while working in the field, clasped his head firmly, declaring that some one had struck him, and complaining of pain in the head. He lost in a few hours all power of speech, but could write, and in that way make himself understood; pupil was dilated; conjunctiva conjested; voluntary motion slow and uncertain; tongue could only be protruded very slowly and considerably to one side; pulse slow and full; respiration labored; at times a remarkable tonic spasm commencing upon one side and gradually extending over the whole system, would fix and hold for several seconds every muscle; respiration, and as far as could be discovered, the circulation would for the time cease, the patient being immovably fixed, often times in very unnatural position; at length the spasm would gradually relax, a deep inspiration would follow, and a profound sleep or coma would succeed, from which he could not be aroused, but which would pass away, leaving him as before, the whole process lasting half an hour or more.

In a few weeks he visited this city, and while here there was sudden increase of the paralysis of left side. After again returning to his home he was attacked by some acute disease of the throat, which made him very sick, as reported by his wife; during this sickness, and after being unable to speak for two months, he was altogether overjoyed to find on awaking one morning, that he could speak quite plainly. This is the point of especial interest, and the reason for relating what would otherwise seem unworthy of mention.

Cerebral effusion is supposed not unfrequently to occur in the course of convulsions, especially puerperal convulsions and epilepsy, the conjestion which is produced, acting as a most efficient cause. Possibly the true character of this attack has not been apprehended, and it may be differently regarded by equally accurate observers. It is difficult to satisfactorily explain the sudden restoration of speech, upon the ground of absorption of effusion, and equally difficult, to account for the phenomena observed, upon any other hypothesis than that of conjestion, producing pressure and loss of function, or effusion of either blood or serum.

*Prof. White* remarked that the subject of serous apoplexy, interested him greatly; recollected visiting, the first year of his medical practice, a man at the Old Eagle Tavern, who had apoplexy. He had been riding for several days and nights; was greatly exhausted, and unquestionably had serous effusion. The patient was bled largely, the practice was approved by his preceptors, and the young physician was highly commended for his energetic and heroic conduct, his active and vigorous treatment; he has long since come to regard it, as exceedingly inappropriate. Dr. W. also spoke briefly of a case treated by Prof. Flint and himself, where they found on arrival at the bed-side of their patient, a practitioner who had been temporarily called, bleeding the patient in a full stream; insisted upon the immediate closure of the vein; regarded it as a case of serous effusion; the patient died. From that time to the present, has made a wide distinction in cases of apoplexy, and desired to call the attention of the profession to the fact, that anemic females were very liable to one form of the disease. Expressed the fear that many physicians were not even now sufficiently aware of the impropriety of indiscriminate bleeding, in cases of this description, or did not carefully discriminate between serous and bloody effusion.

*Prof. White* reported a case of adhesion of the lips of the uterine outlet from inflammation, occasioned by mechanical irritation in an attempt to procure abortion.

M. W. at the Buffalo Lying-in-Asylum, Oct. 17, unmarried; first pregnancy; menstruated last about the first of January, 1861; quickened in May, and expects to be confined the latter part of October. She confesses to having used a large wire or rod of iron, when three or four months advanced in her pregnancy, for the purpose of procuring an abortion. Says "when the wire was introduced some blood followed immediately, and this flowing was soon succeeded by great pain and tenderness in the lower abdominal region, as well as heat and discharge from the genitalia.

*September* 24.—The Sister in charge of the ward informs me that Mary has had pains most of the night, and that they are now quite severe. Upon instituting a vaginal examination, no opening could be found in the lower segment of the uterus. By carrying the fingers carefully up to the junction of the uterus with the vagina posteriorly, and bringing them anteriorly to the pelvic symphysis, there could be found only a slight inequality—a slight elevation or cicatrix, in the lower surface of the uterine tumor. The stehescope applied to the abdomen showed the fœtal heart to be distinctly heard below the umbilicus, upon the left side, and quite low down, and she has all the ordinary indications of having matured her pregnancy. A dose of castor oil was administered, which moved the bowels freely. During the succeeding day and night the pains continued without abatement, and on the 25th, found her quite exhausted with fatigue and sleeplessness. The parts continued without change, except that the uterine neck had been pushed somewhat further down into the pelvis. In the hope that nature, unassisted, would eventually break up the adhesions between the uterine lips and save the necessity of any operative procedure, I directed a large anodyne enema to be given. The pains were lessened, and the patient was able to obtain some refreshing sleep, and take a little nourishment.

During the next three weeks she continued to have recurrences of severe pains, which were permitted to continue until her strength began to fail, when the effort would be made again to control them by anodynes. It required a grain of morphine, and this had sometimes to be repeated, before the gravity of these pains could be so far lessened as to procure sleep. Quinine and beef essence were freely given, and as the pulse and exhaustion did not indicate the danger imminent, interference was postponed from day to day, awaiting an emergency which would demand immediate action. Meantime she was visited by several medical gentlemen, among the number my colleague, Prof. Rochester, who on several occasions made unsuccessful efforts to detect an opening in the lower segment of the uterus.

At length the 16th of October, and the 22d day after the commencement of labor pains, having resolved that something must be done for her relief, I again made a careful vaginal examination. Placing the finger upon what seemed the cicatrix of the os, I made steady, continuous pressure for some time. Upon the recurrence of the pain the pressure was increased until finally the adhesions gave way and permitted the finger to pass into the cavity. She was then left with directions to be sustained by beef essence and brandy until the next morning. Upon examination on the 17th, the orifice was found one and a half or two inches in diameter though yet rigid and cartilaginous. At 3 P. M. the head had cleared the os and descended into the pelvic cavity. The pains being now inefficient and the woman much exhausted, the labor was completed with forceps, the patient being first brought under the influence of chloroform.

She was safely delivered of a male child, weighing 10 lbs. the skull firmly ossified, and presenting all the appearance of a child a month old. From this time, nothing unusual occurred, the woman convalesced rapidly, and both mother and child are now ready to be sent out of the Asylum.

This case suggests several points of interest. In the first place, there can be, in my mind, little doubt that the occlusion was occasioned by the inflammation, superinduced by the mechanical efforts to procure abortion. How often this danger may be incident to the reprehensible, (though truth demands we should add not unfrequent,) resort to such means for procuring abortion, it would be difficult to estimate.

Not the least interesting point illustrated by this case is the absence of danger attending delay in the first stage of labor. For more than three weeks was this woman scarcely free from pain for a moment, and then only partially quieted by large doses of opium, and much of the time she was suffering severely, and yet her labor terminates safely to both mother and child. Had the head descended into the pelvic cavity and entered fully upon the second stage of labor, it could not have been delayed one fortieth part of that time, without endangering the integrity of the maternal tissues lining that cavity, and threatening the life of the child from pressure upon the head.

It will be remembered by several of the members of this Society, that some years since I reported two cases of complete occlusion of the uterus. One of these occurred in the practice of Dr. Charles H. Wilcox, and the other was under the care of Dr. Baker. In both, surgical interference was rendered necessary, and successfully resorted to at a much earlier period. The instance just related will scarcely form a precedent which it would be safe in all cases to follow. It will however, always be well to delay any incisions through the occluded neck, in the hope that nature herself, may open the way, until symptoms are present requiring our interference.

*Dr. White* called the attention of the Society to the use of "sponge tents," for dilating stricture of the rectum. He had recently resorted to them in the following case, with very gratifying results, and hoped upon further trial by the members of the Society, they might be found useful in many cases of simple stricture, non-malignant in character, and situated near the anus.

Mrs. W. aet 26, was married at 16, had her first child after a severe labor at 19 years of age. Her convalescence was protracted, during which she suffered from pelvic abscess which left her with *fistula in ano.* Two years subsequently she was operated upon for fistula by Dr. Bigelow of Boston, with entire relief. Since that time she has been troubled with difficult defecation, growing more and more annoying until now, the fecal matter can be expelled only by great effort, and after taking large warm water injections. When the stools are so consistent as to be moulded by the stricture, they are not larger than a "pipe stem," and are expelled with the utmost difficulty. Her general health has become gradually impaired during the last year, and she now consults me for relief.

Upon making a digital examination of the rectum, a soft stricture is found two or two and one half inches above the sphincter and just at the point where the incision seems to have been made for the cure of the fistula. The point of the finger could not be inserted into it by any amount of pressure which the patient could endure. Being in the habit of using sponge tents for dilating the neck of the uterus, it occurred to me, that they might be made available for the purpose of dilating rectal contractions.—Accordingly I inserted a large and long uterine tent; as far as possible into the stricture; this tent was not more than three lines in diameter; directed her to sit down in a warm bath. In thus applying warm water to the outer extremity of the tent, instead of waiting for the purulent fluids from above to be first absorbed and enlarge the upper end, the difficulty of withdrawal was obviated. The result was highly satisfactory. The tent gradually expanded from below, and so gently, that, soothed by the warm water, she was able to permit its complete expansion before she withdrew it. The relief was so manifest, I was induced the second day after to insert a tent four or five times its diameter, and very firmly made. This, like the preceding, was permitted to completely unfold before removal, and considerably increased the dilitation of the stricture. Two days subsequently a tent measuring a little more than half an inch where it occupied the stricture, was in the same manner introduced and soaked with water until fully expanded. Some difficulty was experienced by the patient in withdrawing the tent on this occasion, in consequence of the part of the tent situated above the stricture becoming saturated and fully expanded. The finger could now be easily passed completely through the stricture, and the indurated edges were found greatly softened and absorbed. She was now furnished with a rectum bougie, the diameter of which is more than an inch at the point where it engages in the stricture, and directed to insert it frequently to prevent its recontraction. The patient believes herself completely relieved of the local difficulty, and her general health is greatly improved.

*Dr. White* closed his remarks by expressing the hope that other members of the Society would be induced to make trial of the tents in analogous cases, as he was aware that nothing could be established by a single case, but confessed to great confidence in the success of the plan here adopted.

*Dr. Miner* thought the means adopted by Prof. White for dilating stricture of the rectum ingenius, philosophical and efficient, certainly in the hands of Dr. White. Had never been greatly pleased with the sponge as a dilating instrument; could not think that the expanding force of a dry sponge from the absorption of moisture could be very great; had never been able to introduce sponge with sufficient force without its bending or breaking; had often thought a conical instrument made of soft rubber more cleanly, durable and efficient. Spoke of the readiness and rapidity with which absorption of the fibrinous deposit, which is the usual cause of stricture, would sometimes take place, especially in the urethra, when an instrument was passed down so as to press somewhat upon it, even though no dilating process is instituted. Could not offer any experience, in the treatment of stricture of the rectum since it was one of the rarest forms of disease, disconnected with scirrhus, and based his remarks upon his observation in stricture of urethra, which had many features in common with it. Stricture of the rectum treated successfully by whatever means adopted, is of interest and importance, such disease being generally very intractible it being difficult to obtain complete and permanent relief.

*Dr. Kempson* would quite agree with the remarks of Dr. Miner, that the relation of this case in which the ingenius application of sponge as a dilating medium in the stricture of the rectum, might result in the formation of a very useful instrument by bringing to its aid India rubber. Thought a hollow elastic tube, filled with dry sponge, cut to the proper size, would be easier introduced than sponge alone, and moisture would compel the tube to expand. Dr. K. also related a case of severe injury of the rectum, observed by him while a student of medicine in Kidderminster, England. A robust young man fell backwards upon an inverted blacking pot, while in the act of relieving his bowels, with such force as to completely drive it within the rectum beyond the reach of the fingers; the pot was one and a half inches at the top and one inch at the bottom in diameter, and about three inches high, every effort to extract having failed, the pot was broken and the pieces very carefully removed. The next morning violent inflammation having set in, he was bled largely, and at intervals as many as 60 leeches were applied, however, he sunk rapidly, and died, in fifty hours after the accident. Dr. K. would ask if it had not occurred to any one present, that cases of imperforate os-uteri were sometimes produced intentionally by the manipulations of physicians, who do not hesitate to depart from the legitimate object of our art, and in order to pander to the lascivious desire of their patients, and allow them freely to indulge in illicit intercourse, have recourse to various methods to bring on adhesive inflammation of the womb and produce a complete closure of the orifice. This is done under the guise of healing ulceration of the neck of the womb, &c., and these cases constitute the great bulk of the practice of a Canadian practitioner who enjoys a high reputation among the best classes in Canada; at least so he is told. If it is so, hoped that these remarks might reach his ear, or his eye, that he may desist from such diabolical practice.

*Dr. Miner* presented (at the request of a friend who had suggested it,) the most economical tourniquet yet invented; it consisted of a plain strap of India rubber, an inch in width, and half a yard in length; its application was explained, and this very simple instrument represented as possessing many advantages as a tourniquet, especially adapted to some of the emergencies in military practice. Its exceeding economy would make it available for every soldier, who might in this way have always at hand efficient means for temporarily suppressing dangerous hemorrhage; again its usefulness in the hands of the medical cadets in time of action, used for tourniquet or bandage, or as part of the outfiit of ambulance carriages was very favorably represented by Dr. M. who summed up its advantages as follows:—Can be more readily and rapidly applied than any other dressing; can be adjusted to suppress bleeding with the greatest certainty, each turn adding as much to the compression as is drawn upon the free extremity; it is compact, cleanly, durable, and in the hands of a good assistant could be very well used as an instrument for surgical operations, though its chief advantages are for more common and temporary uses.

Voted to adjourn until Tuesday Evening, December 3d.

J. F. MINER, Secretary.

